# Data-driven classification and explainable-AI in the field of lung imaging

**DOI:** 10.3389/fdata.2024.1393758

**Published:** 2024-09-19

**Authors:** Syed Taimoor Hussain Shah, Syed Adil Hussain Shah, Iqra Iqbal Khan, Atif Imran, Syed Baqir Hussain Shah, Atif Mehmood, Shahzad Ahmad Qureshi, Mudassar Raza, Angelo Di Terlizzi, Marco Cavaglià, Marco Agostino Deriu

**Affiliations:** ^1^PolitoBIOMed Lab, Department of Mechanical and Aerospace Engineering, Politecnico di Torino, Turin, Italy; ^2^Department of Research and Development (R&D), GPI SpA, Trento, Italy; ^3^Department of Computer Science, Bahauddin Zakariya University, Multan, Pakistan; ^4^College of Electrical and Mechanical Engineering, National University of Sciences and Technology, Rawalpindi, Pakistan; ^5^Department of Computer Science, Commission on Science and Technology for Sustainable Development in the South (COMSATS) University Islamabad (CUI), Wah Campus, Wah, Pakistan; ^6^School of Computer Science and Technology, Zhejiang Normal University, Jinhua, China; ^7^Zhejiang Institute of Photoelectronics & Zhejiang Institute for Advanced Light Source, Zhejiang Normal University, Jinhua, Zhejiang, China; ^8^Department of Computer and Information Sciences, Pakistan Institute of Engineering and Applied Sciences (PIEAS), Islamabad, Pakistan; ^9^Department of Computer Science, Namal University Mianwali, Mianwali, Pakistan; ^10^Department of Computer Science, Heavy Industries Taxila Education City (HITEC), University of Taxila, Taxila, Pakistan

**Keywords:** chest X-ray images, deep learning models, ensemble methods, traditional machine learning, pretrained deep learning models, feature extraction, explainable-AI

## Abstract

Detecting lung diseases in medical images can be quite challenging for radiologists. In some cases, even experienced experts may struggle with accurately diagnosing chest diseases, leading to potential inaccuracies due to complex or unseen biomarkers. This review paper delves into various datasets and machine learning techniques employed in recent research for lung disease classification, focusing on pneumonia analysis using chest X-ray images. We explore conventional machine learning methods, pretrained deep learning models, customized convolutional neural networks (CNNs), and ensemble methods. A comprehensive comparison of different classification approaches is presented, encompassing data acquisition, preprocessing, feature extraction, and classification using machine vision, machine and deep learning, and explainable-AI (XAI). Our analysis highlights the superior performance of transfer learning-based methods using CNNs and ensemble models/features for lung disease classification. In addition, our comprehensive review offers insights for researchers in other medical domains too who utilize radiological images. By providing a thorough overview of various techniques, our work enables the establishment of effective strategies and identification of suitable methods for a wide range of challenges. Currently, beyond traditional evaluation metrics, researchers emphasize the importance of XAI techniques in machine and deep learning models and their applications in classification tasks. This incorporation helps in gaining a deeper understanding of their decision-making processes, leading to improved trust, transparency, and overall clinical decision-making. Our comprehensive review serves as a valuable resource for researchers and practitioners seeking not only to advance the field of lung disease detection using machine learning and XAI but also from other diverse domains.

## 1 Introduction

In modern healthcare, radiology imaging has emerged as a primary tool for the early detection of various diseases (Beets-Tan et al., 2020). Multiple modalities of radiological imaging are employed, including X-ray, Computed Tomography (CT), Magnetic-Resonance Imaging (MRI), Positron Emission Tomography (PET), and Ultrasound (US). Among these, chest radiography is particularly notable for its widespread use in achieving precise and rapid diagnoses (Abhisheka et al., 2023; Hussain et al., 2022; Philip et al., 2023). Radiologists, in the context of chest diseases, predominantly rely on X-rays and CT scans (Hall and Giaccia, 2006).

In most radiological imaging chest diseases, Pneumonia is an acute infection affecting the alveoli and distal airways of one or both lungs. It causes narrowing or closing of the airways due to inflammatory cells and fluid deposition in the alveolar sack of the lungs. It is a common and potentially lethal illness, affecting more frequently susceptible individuals, especially children of < 5 years of age, accounting for 15 % of all deaths caused in this year's groups. Babies born too early (prematurely) face higher health risks because their organs are still developing. The younger the baby is, the higher the chance of getting lung infection (Prodanovic et al., 2024). The infectious agents can be viruses, bacteria, or fungi with a great geographical prevalence and outcome variations, among developed and low-and-middle-income countries. Yearly over millions of people are being infected with this virus. Early diagnosis is crucial for the successful treatment process. Generally, the disease can be diagnosed from chest X-ray images, but it is challenging due to unclear appearances and confusion with other diseases. There is an increasing effort to reach the poor community, in term of helping strategies recognizing and managing pneumonia (Rudan et al., 2004; Torres et al., 2021; Bhutta, 2007; Nelson et al., 1995; UNICEF, 2022; Franquet, 2001). In Africa, a significant shortage of healthcare workers poses a critical challenge, with an estimated deficit of 2.4 million doctors and nurses. Numerous studies have highlighted the severe scarcity of medical professionals and other healthcare resources across the continent (Narasimhan et al., 2004; Naicker et al., 2009). Due to critical health issues, the early detection of this disease, especially in neonates, is very important as it interferes with brain growth development and leads to other visual and hearing impairments including heart diseases (Maeda et al., 2023; Yildirim and Canayaz, 2023; Prodanovic et al., 2024).

Considering AI as a promising tool in medical imaging, AI can improve pediatric pneumonia diagnosis in the early stages (Yoon and Kang, 2024; Bal et al., 2024). In this scenario, specific kinds of challenges arise when working with pediatric chest X-ray datasets. Firstly, the available data scarcity, as compared to adult datasets, can hinder the development of robust trained AI models. Secondly, pediatric X-ray images reveal diverse anatomical development structure as compared to adults. Thirdly, it is also very challenging to acquire high quality images from uncooperative young ages patients and will result in various noise factors and artifacts caused by positioning and breathing patterns. Due to this, it will lead to more complicated analysis. Finally, ethical rules and regulations come into play even more critically when dealing with pediatric patients as compared to adults. Strict anonymization protocols and parental consent procedures are very important to ensure data collection and further utilization (Singh, 2024; Candemir and Antani, 2019; Ciet et al., 2024). Considering all these challenges, the collaborative effort of data collection and the development of robust AI powered diagnostic tools require standard image normalization techniques and strict anonymity rules and regulations for early diagnostic of pneumonia in pediatric patients.

Over the last 20 years, it is noted that chest infection has decreased. New conjugate vaccines for diagnosing *Haemophilus influenzae* type b and *Streptococcus pneumonia* helped to decrease in radiologic, clinical and immense pneumonia cases have reduce hospitalization (Le Roux and Zar, 2017). In 2011, the western pacific region, recorded that 61,900 yearly deaths are due to pneumonia in < 5-year-old children (Nguyen et al., 2017). According to world health organization (WHO), all these extraordinary reductions in deaths, pneumonia is one of biggest killer of children < 5 years old (Chavez et al., 2015).

Today, chest X-ray is used to show the presence of different lung diseases in humans. Experienced radiologists can assume the probability of chest disease in humans after checking the x ray images. Notably, in 2019, hospitals in Italy and the UK primarily employed radiography imaging to diagnose coronavirus patients (Fields et al., 2020). In some regions of the world, there is a limited amount of medical equipment and a shortage of doctors and experienced professionals who can effectively interpret chest X-rays. The most often experts of radiology examine the two-dimension (2D) chest x-ray images to clarify the pneumonia bacteria or virus instead of three-dimension (3D) chest structure (Mahomed et al., 2020).

The ease of obtaining radiographic images, however, also poses a significant challenge, as a single radiologist may need to evaluate as many as 100 images daily through radiography imaging [3]. Therefore, medical image analysis is a time taking process to reach chest disease diagnosis. In this scenario, information technology (IT) has a cardinal importance and is assisting the radiologists. It has also demonstrated itself as a backbone in other medical fields too. Further, health IT is administering the information of different kinds of disease using computational knowledge and its progress. The promising capability of health IT in decision making is a lot more as compared to a human. In this way, health IT can provide more better assistance in diagnosing various diseases to all over the world's medical community such as for acute coronary syndrome (D'Ascenzo et al., 2021), cardio-vascular risk (Navarini et al., 2020), and so on. Moreover, the most tremendous things in IT is the available data on the internet and that anyone can access at any time (Bohr and Memarzadeh, 2020). In parallel, advancements in artificial intelligence (AI) has led the medical field to consider AI as a promising tool for tackling complex biomarkers and supporting medical researchers in comprehending the progression of various diseases (Xing et al., 2020).

In this comprehensive review, we have examined and compared 20 computer-aided systems designed to assist clinicians in diagnosing pneumonia. Our exploration began with an in-depth analysis of publicly available datasets. We also provided detailed insights into various pre-processing techniques to aid researchers related to the available data. Furthermore, we conducted an extensive discussion on the various machine learning and deep learning algorithms that have been predominantly utilized in recent research efforts. Our aim is to highlight the best models for a reliable and versatile tool useful in the middle- and low-income communities.

The paper is divided into several sections. Section 2 focuses on the dataset and its modality. Section 3 describes the data preprocessing and balancing techniques, and Section 4 discusses the different performance measures used in the studies. Section 5 elaborates numerous techniques proposed in this domain and section 6 provides a detailed comparative analysis and discussion of all techniques used for chest diseases' classification. Section 7 discusses progress and challenges in research works related to chest diseases. Finally, Section 8 concludes the review work.

## 2 Available training datasets

In contemporary times, a variety of datasets for lung diseases, complete with labels, have become accessible for training diverse machine learning and deep learning algorithms. These datasets have consistently served as standard references in numerous research endeavors, facilitating the comparison of results achieved through different techniques. Considering the wealth of available datasets, it is evident that they provide ample resources for training, validating, and testing machine learning and deep learning algorithms, including the creation of holdout datasets for evaluation. In our study, we focused on several benchmark datasets containing chest disease data and reviewed pneumonia experiments on these datasets, as mentioned below.

### 2.1 OCT-CXR

This dataset (Kermany et al., 2018) comprises 5,863 chest X-ray (CXR) scans from children, organized into three partitions: training, validation, and testing. The CXR images were taken meticulously and carefully selected from pediatric patients treated at the China Medical Center. All X-rays were taken as part of routine checkups.

### 2.2 CHEST X-ray 14

This dataset (Wang et al., 2017) was collected from the clinical picture archiving and communication system (PACS) and consisted of several frontal chest X-ray images. The dataset contains 112,120 front-facing chest X-ray images, which were subdivided into 14 classes. Each class represents a disease that could be identified from radiology reports.

### 2.3 MIMIC-CXR-JPG

This dataset (Johnson et al., 2019) is the largest publicly available chest X-ray dataset. The dataset was gathered by the USA Medical Center from 2011 to 2016. More than 35,000 chest X-ray images that represent 14 different chest diseases, including pneumonia and normal cases, were obtained.

**Figure 1:**
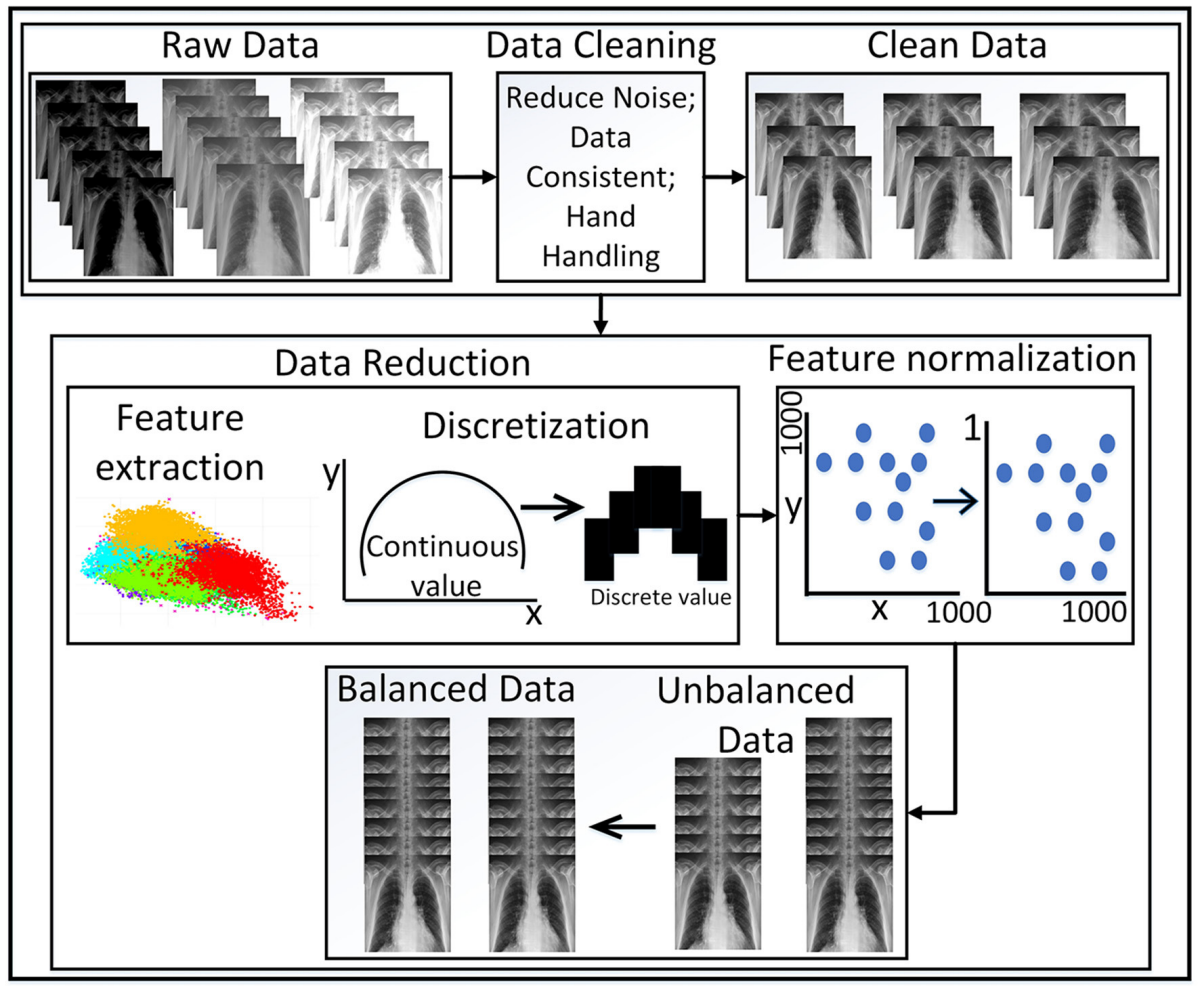
Illustration of the general preprocessing steps.

Additionally, Table 1 provides an overview of the chest X-ray datasets that were used in previous studies for pneumonia classification. Some datasets were custom designed by compiling for pneumonia classification tasks, while others included multiple chest diseases, including pneumonia by default. The general steps that were taken by most of the strategies to make the data ready for classification have been described previously Figure 1 and have also been employed by many related studies (Benhar et al., 2020). The dataset with the highest number of pneumonia cases is called CXR, but in terms of image count, the MIMIC-CXR-JPG dataset is larger due to its inclusion of multiple chest diseases.

**Table 1:** Comparison of datasets for pneumonia cases.

**Dataset**	**No. of images**	**Description**
OCT-CXR	5,856	Pneumonia binary cases either pneumonia or normal class
Chest X-ray14	108,948	1,500 pneumonia cases. The dataset includes 8 different chest diseases
MIMIC-CXR-JPG	377,110	Enormous dataset consisting of 65,079 number of pneumonia patients
PneumoCAD	156	Only 78 pneumonia cases exist
COVID-19	219	Comprises samples of three classes COVID-19 positive, normal, and viral pneumonia
PediCXR	9,125	Consists of 481 pneumonia cases and remaining dataset is divided into other 14 different labels
CheXpert	224,316	Having 14 different classes while pneumonia cases are 4,576
NIH Chest X-ray	112,120	Comprises 1,062 pneumonia images and remaining other pathologies are divided into 7 classes

### 2.4 PneumoCAD

This dataset (Oliveira et al., 2008) comprises pneumonia presence (PP) and pneumonia absence (PA) images based on children scans. All these findings were confirmed by two radiologists. These authors interpreted the chest radiography images according to the WHO instructions. These radiography images had a resolution of 1,024 x 768 pixels and an 8-bit grayscale. A total of 156 X-ray images were inspected by radiologists; 78 images were from pneumonia patients, and the remaining 78 were labeled as normal.

### 2.5 COVID-19

The COVID-19 dataset (Cohen et al., 2020) consists a of total 219 chest X-ray images. It comprises three classes COVID-19 positive, normal, and viral pneumonia. Further, each image varies in size, typically around 1,024 x 1,024 pixels, though this can vary due to different sources and preprocessing steps. The dataset is organized into three classes, each representing a different health condition, providing a balanced set of examples to train and evaluate machine learning models for accurate diagnosis and classification of COVID-19 and related respiratory conditions.

### 2.6 PediCXR

This dataset (Pham et al., 2023) is a very valuable resource for training and evaluating AI models for pediatric chest X-ray images. It consists of 9,125 X-ray scans and gathered at major pediatric hospital in Vietnam between 2020 and 2021. Each image possesses a different pixel range, but most cases have more than 1,000 pixels in both x and y dimensions. The dataset is enriched with 36 critical findings and 15 distinct disease categories. This comprehensive labeling scheme allows researchers to train AI models for various tasks, from pinpointing specific abnormalities to making broader diagnoses in pediatric chest X-rays.

### 2.7 CheXpert

CheXpert (Irvin et al., 2019) is a larger collection, containing 224,316 chest radiographs from adult patients. While image sizes aren't explicitly stated, they likely adhere to medical imaging standards, potentially ranging from 512 x 512 pixels to 1,024 x 1,024 pixels. In addition, CheXpert includes labels for 14 different pathologies.

### 2.8 NIH Chest X-ray

The NIH Chest X-ray dataset (Wang et al., 2017) is containing 112,120 frontal and lateral chest X-rays from adult patients. Image sizes are likely similar to CheXpert's. However, the dataset utilizes a simpler labeling scheme, focusing on the presence or absence of eight common findings, including pneumonia.

## 3 Preprocessing

The purpose of preprocessing is to improve the quality of images by reducing variations and eliminating any discrepancies that may cause complications during the processing stage, thereby reducing the consumption of resources (Tripathy and Swarnkar, 2020). Preprocessing achieves three main objectives, namely noise removal, contrast enhancement, and brightness and color correction. To remove noise, filters such as mean and median filters, and Gaussian low-pass filtering are widely used. Morphological techniques are also used for image information enhancement purposes (Courtenay et al., 2020). Contrast stretching techniques and histogram equalization techniques have been widely used for contrast enrichment. For brightness, color correction, and color standardization techniques have been used also such as Gastal and Oliveira (2012).

Furthermore, standard x-ray images generally have a dimension of (3,000 x 2,000) pixels, which is quite large and contains unnecessary information. These large images require high storage space and powerful hardware for analysis. To save time and obtain better results, many publicly available datasets have resized their images from their original size. For example, the Chest X-ray 14 dataset has reduced the image sizes to 512 x 512 pixels, while the MIMIC-CXR-JPG and OCT-CXR datasets have image sizes of (2,048 x 2,048) and (1,024 x 1,024) pixels, respectively. In our studies, we have also emphasized the importance of a balanced experimental dataset for training, validation, test sets. An unbalanced data set can lead to biased classifications toward the majority image set. Therefore, data balancing techniques are used to prevent such biased classification results. Table 2 illustrates some traditional and deep learning data balancing techniques along with their descriptions.

**Table 2:** Data balancing techniques.

**References**	**Technique**	**Description**
Wang and Lu (2018)	Mean Square Error (MSE)	Sum square error of every sample class wise and then calculated their average
Wang et al. (2016)	Mean False Error (MFE)	Calculate the loss based on average error
Baltruschat et al. (2019)	Manually sampling	Samples randomly duplicates in minority class while samples randomly removed from majority class
Feng et al. (2019)	Synthetic Minority Oversampling Technique (SMOT)	Oversampling method

## 4 Performance metrics

The performance measures (Michelucci et al., 2021) for pneumonia detection and classification depend upon various factors. These factors are discussed below:

- True positives (TPs) that identified the correct pneumonia cases from the training data.
- True negatives (TNs) were those that identified normal cases from the training data.
- False positives (FPs) that incorrectly identified pneumonia cases.
- A false negative (FN) was used to identify the wrongly normal cases.

The following equations represent the performance metrics used to evaluate the robustness of the models: accuracy (1), sensitivity (2), specificity (3), and precision (4).


(1) $$\begin{array}{l} Accuracy\;\left(acc\right)\;=\frac{TP+TN}{TP+FN+TN+FP} \end{array}$$

(2) $$\begin{array}{l} Recall/Sensitivity\;\left(rec/sen\right)=\frac{TP}{TP+FN} \end{array}$$

(3) $$\begin{array}{l} Specificity\;\left(spe\right)=\frac{TN}{TN+FP} \end{array}$$

(4) $$\begin{array}{l} Precision\;\left(pre\right)=\frac{TP}{TP+FP} \end{array}$$

## 5 Classification methods

**Figure 2:**
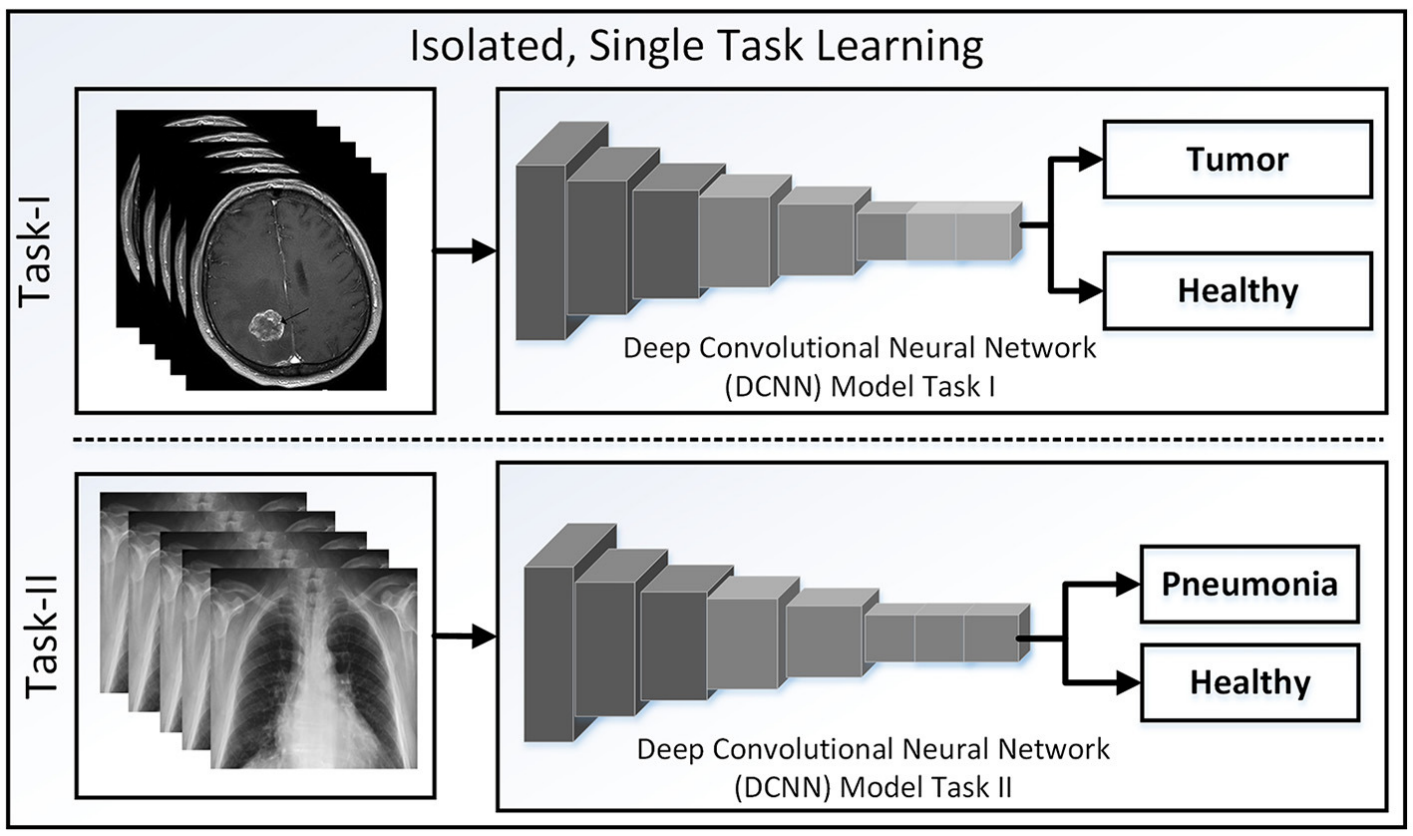
Basic concept of traditional learning where brain tumor images are taken from Chakrabarty (2019).

Over time, machine learning approaches, a general concept, have been shown Figure 2 to be implemented in numerous studies (Hameed et al., 2020; Javaid and Haleem, 2020), significantly assisting in the medical field. Even today, machine learning is useful in the medical field and helps researchers continue to address novel medical issues. A number of relevant studies on pneumonia have been published in the subsequent subsections based on CXR datasets. These used CXR datasets are frequently used in research as a standard dataset for comparison with the results of other studies. In our review, we adhered to the same framework.

### 5.1 Machine learning models

In traditional machine learning, researchers have applied feature extraction algorithms to images to address specific problems. In this context, datasets have been analyzed for only a single domain at a time. More complexity and more time are required to obtain results from these algorithm techniques.

Oliveira et al. (2008) proposed a machine learning technique that classifies X-ray images as pneumonia or normal cases. On the images, they employed the Haar wavelet transform feature extraction computer vision technique. The KNN algorithm was subsequently used, where K was 15, to train and classify the model on the extracted features. As a result, they reported an accuracy of 97% compared to that of other previously published feature extraction studies in this domain. The results showed that this method achieved good accuracy due to the application of relevant feature extraction techniques.

Sousa et al. (2013) compared different machine learning techniques and evaluated their results. In this work, three random sets of 40 images were used for training, and 15 images were randomly selected for the testing phase. For features, different texture-based feature extractors based on the Haar wavelet transform (Yu et al., 2023) were used to extract features for classification tasks (Choras, 2007; Guido, 2018; He et al., 1987). Three machine learning techniques, namely, SVM, KNN (K = 9), and naïve Bayes (Kumar et al., 2019; Zheng and Ding, 2020; Kaur and Oberoi, 2020) classifiers, were applied to these extracted features and achieved accuracies of 77, 70, and 68%, respectively.

Yao et al. (2011) proposed an automated intelligent system that identifies five different chest diseases, including pneumonia. They used 40 CXR images and applied machine learning to texture analysis. Their extracted feature vector contains 25 texture features (mean and variance features from histogram statistics, energy and correlation features from a co-occurrence matrix, etc.) for every image in the dataset. These extracted features were further passed to train and test the SVM model, which yielded 85% accuracy in the pneumonia detection task.

### 5.2 Deep learning models

DL has pushed research work in all fields by limiting the use of standard handcrafted mechanisms of detection and classification (Bhandary et al., 2020). Deep learning models are composed of many layers connected to each other and have designated functions. All these layers are used for their designated function in the respective successions. Deep learning works best on a large number of training sets that are not easy to obtain in the biomedical field because it is supervised (Suzuki, 2017; Khan et al., 2021).

The dependence on human operators for the diagnosis of pneumonia is not suitable because it requires expertise and experience. Therefore, the domain of deep learning is currently being adopted to make systems fully self-driven. This approach provides better results, fewer miscalculations and fewer chances of failure (Sun et al., 2018). Deep methodology is the latest innovation inside the domain of machine learning. It is constantly being pruned and enhanced daily, so much research is being conducted on this topic. We can expect more work in the field of cell diagnosis and disease classification soon, which will involve the use of deep networks.

**Figure 3:**
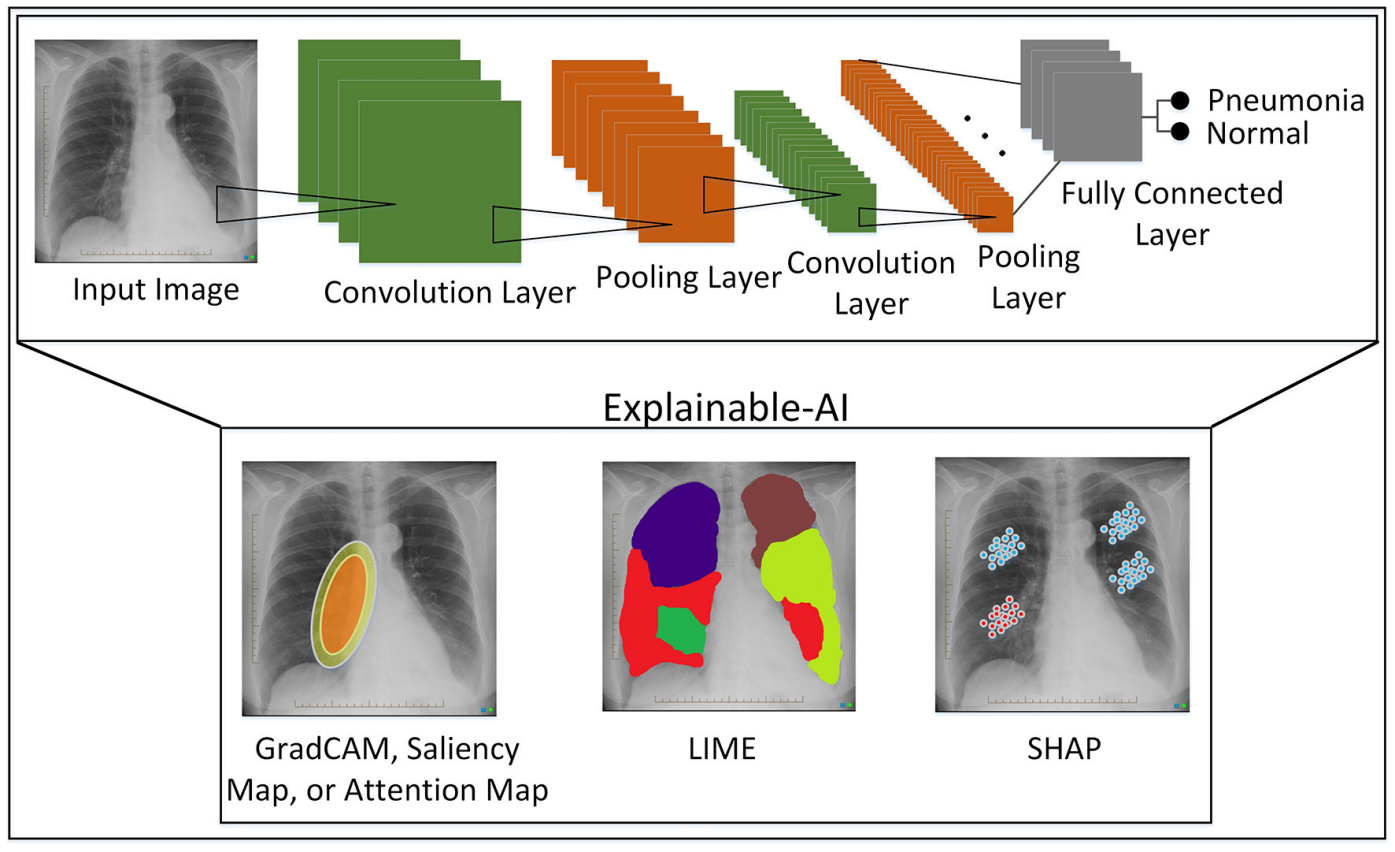
Convolutional neural network (CNN) model with XAI techniques.

CNNs have been widely used to improve classification and segmentation tasks. In CNN, the convolutional layer helps to extract multidimensional features from the input image. The applied weighted distributed technique helps to reduce the complexity of the network (Albawi et al., 2017). Architecturally, CNNs are simply feedforward artificial neural networks. The general structure of the CNN model based on several blocks is shown in Figure 3. This strategy is widely employed in various studies, such as those of Yamashita et al. (2018) and Acharya et al. (2017), who adopted a similar approach to provide a comprehensive overview. In addition to classification tasks, explainable-AI (XAI) techniques are proposed for revealing black boxes, such as the following:

- **Local Interpretable Model-Agnostic Explanations (LIME):** Ribeiro et al. (2016) works by developing local interpretable models around a specific target, and the developed model is used to explain why that prediction was made.
- **Shapley Additive exPlanations (SHAP):** This technique (Lundberg and Lee, 2017) explains the black box model by assigning a shap value to each feature that tells the feature's contribution to the model prediction.
- **Saliency maps:** This technique (Zeiler and Fergus, 2014) unveils black boxes on images by highlighting the most important features in an input image.
- **Gradient-weighted Class Activation Mapping (Grad-CAM):** Grad-CAM (Selvaraju et al., 2016) works on the saliency map technique for CNNs. The gradient of the model's output is computed with respect to the input image, and gradient maps are developed.
- **Activation maps:** This technique (Zeiler and Fergus, 2014) produces images that possess the activations of neurons in a CNN. This technique is helpful for understanding the internal operation of CNNs.

In the upcoming subsections, we have mentioned custom-designed deep models and transfer learning-based models that researchers have employed in their work regarding imaging-based pneumonia disease detection.

#### 5.2.1 Customized CNN models

Stephen et al. (2019) proposed a CNN with four convolutional layers and two dense layers. They proposed a model trained on the OCT-CXR dataset. They split the dataset for training and testing on their own dividing threshold from the original dataset and used data augmentation techniques to increase the performance of the model up to 93.7% testing accuracy, as reported in their work.

Jain et al. (2020) worked to classify pneumonia and non-pneumonia chest X-ray images. The author used six models in this research to compare the performances of these models. The first 2 models were constructed by using several convolutional layers, and the other four models were pretrained (VGG-16, VGG-19, ResNet-50 and Inception-V3). All the models were trained and tested on the CXR dataset. The best classification result of the second model, which was based on three convolutional layers, achieved 92.31% accuracy compared to the other models.

Siddiqi (2019) proposed a CNN model that used an 18-layer convolutional neural network and trained it using the OCT-CXR dataset following a dataset with 80% disturbance in the training set and 20% disturbance in the testing set. The proposed technique achieved accuracy, specificity, and sensitivity of 94.3, 86, and 99%, respectively. This study showed that these methods improved the classification accuracy by 1.6%, but on the other hand, they had lower specificity than did the state-of-the-art methods.

In addition to the research in the same domain, Labhane et al. (2020) obtained better results from different neural network models using custom-CNN and transfer learning techniques on three renowned models, namely, Inception-V3, VGG-19, and VGG-16. In addition to the results of other studies in the same domain, better results were obtained from different neural network models trained with custom-CNNs and transfer learning techniques on three renowned models, namely, Inception-V3, VGG-19, and VGG-16. The dataset (OCT-CXR) used in these models included 2,992 pneumonia images and 2,972 chest X-ray images. Using augmentation techniques, the training data increased, which helped in obtaining the most promising performances, ~97% accuracy, for customized and all the renowned deep networks trained in this work.

Similarly, Liang and Zheng (2020) proposed a custom 49 convolutional layer residual model and renowned the VGG-16, DenseNet-121, Inception-V3, and Xception models for transfer learning. In this work, the OCT-CXR dataset was used to classify patients into binary response groups, such as normal vs. pneumonia. This work achieved 90.5, 74.2, 81.9, 85.3, and 87.8% accuracy, respectively, as mentioned previously. Liang and Zheng also concluded that their customized model performed better in classification than did transfer learning.

Saraiva et al. (2019) shows comparative research between conventional (multilayer perceptron) and custom-designed deep learning models on the OCT-CXR dataset considering binary classification. The experimental data had two classes consisting of 5,840 images with infected and non-infected traits. During training, a cross-validation technique was used to validate the models. Based on the performance, the deep learning models performed very well, with 94% accuracy, whereas the conventional neural network model achieved 92% accuracy.

Yi et al. (2023) designed an intelligent system for the detection of normal and pneumonia diseases using chest X-ray images. The main objective of this study was to illustrate the strength of deep neural models on image-based data. In the first step, they acquired a publicly available OCT-CXR dataset. In the next step, they applied data augmentation and preprocessing steps such as rescaling, rotation, width-height shift, shearing, zooming, horizontal-flipping, and filling to prevent bias in the data sample class. For feature learning and extraction, they designed a proposed DCNN model with 42 conv layers and 2 dense layers. After the training step, they utilized the trained deep model as a feature extractor to extract robust features from the training and validation datasets and then applied a supervised machine learning classifier for pneumonia classification. For the performance evaluation of machine learning models, standard parameters such as accuracy, specificity, sensitivity, and the F1-measure were acquired. The proposed methodology achieved 98.02% training accuracy and 96.09% test accuracy scores.

Akbulut (2023) introduced an innovative and robust algorithm, the ACL model, which was designed as a customized deep learning architecture. This model was purposefully combined with attention and LSTM models and CNN frameworks to accurately classify patients into distinct categories: healthy, COVID-19, and pneumonia. To enhance the performance of this approach, crucial features and patterns present within chest X-ray images were emphasized. This was achieved through the application of the marker-controlled watershed (MCW) segmentation algorithm, which highlights essential stains and traces vital for accurate classification. Throughout the experimental phase, the ACL model underwent training across varying training–test ratios—specifically, 90–10, 80–20, and 70–30%. The attained accuracy scores demonstrated exceptional performance, recording a perfect accuracy of 100% for the 90–10% ratio while achieving an impressive 96% accuracy for both the 80–20 and 70–30% ratios. These outcomes underscore the adaptability and reliability of the ACL model across different training-test distributions, demonstrating its potential as an effective diagnostic tool for classifying health conditions based on CXR imaging data.

In another work, Kiliçarslan et al. (2023) developed a novel deep learning methodology aimed at pneumonia classification. This study introduced an innovative activation function termed Superior Exponential (SupEx) and conducted comparative evaluations against established activation functions such as ReLU, LReLU, Mish, Sigmoid, Swish, Logish, and Softplus. The study integrated the MNIST and CIFAR-10 datasets to substantiate the efficacy of the proposed SupEx activation function. The findings were subsequently applied to the CNN, which trained and tested pneumonia identification via chest X-ray images. Notably, classification accuracies were achieved, reaching 95.37% for pneumonia detection. This research signifies the introduction of a promising activation function for pneumonia detection, demonstrating its efficacy across both emerging and established benchmark datasets.

#### 5.2.2 Transfer learning models

**Figure 4:**
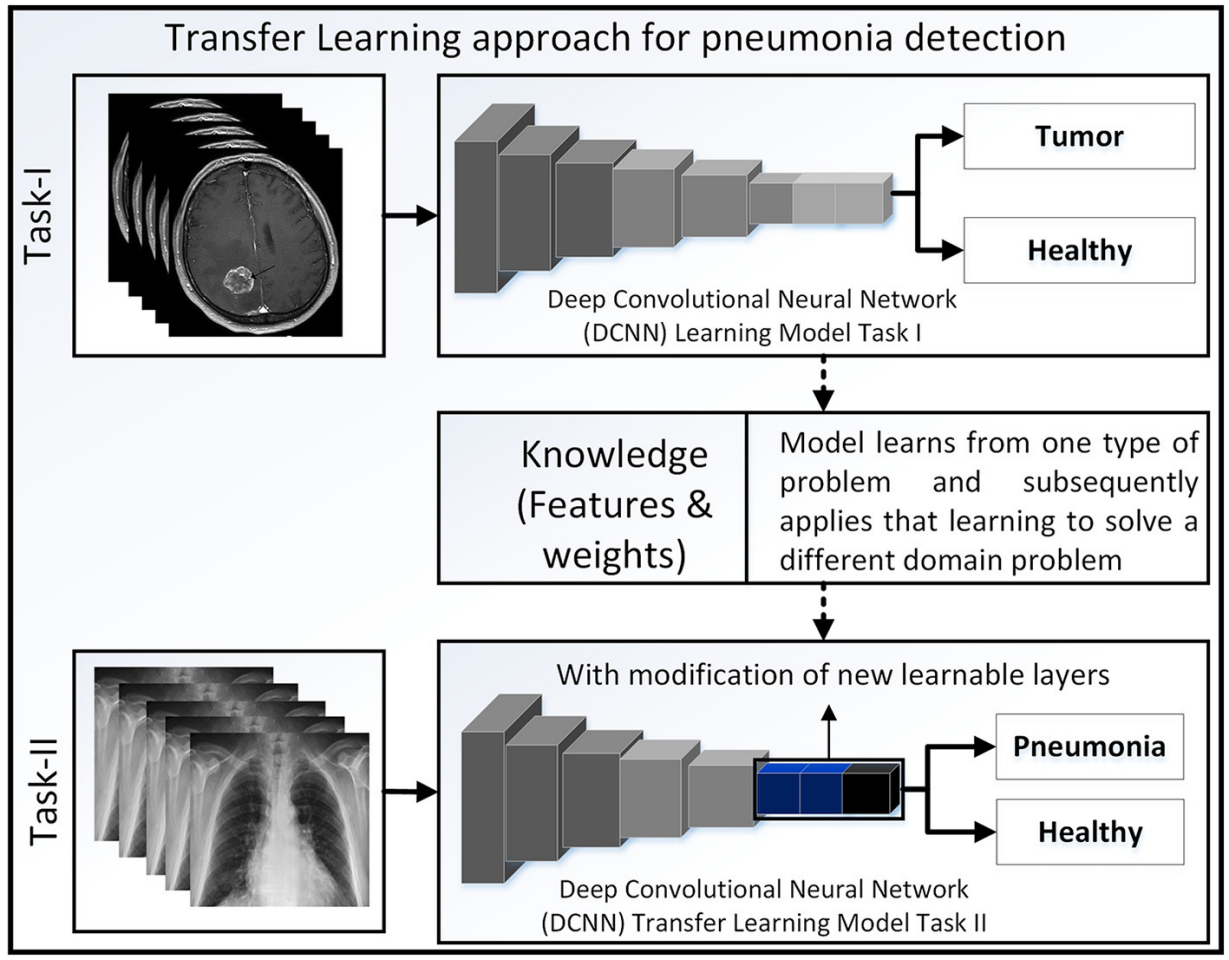
Concept of transfer learning where brain tumor images are taken from Chakrabarty (2019).

Transfer learning is the concept of overcoming individual learning complexities and reusing the knowledge that is obtained from any similar pretrained model. Figure 4 illustrates the fundamental principles of transfer learning, and numerous investigations have explored its application in the classification of lung diseases, as demonstrated in Raghu et al. (2019).

Rajaraman et al. (2019) proposed a customized CNN model and compared their results with those of a VGG-16 retrained model on the OCT-CXR dataset. In this research, an atlas-based detection algorithm was used to detect lung tissue, and a DCNN model was used for classification. In comparison, VGG-16 achieved better performance than did the customized CNN. The author performed two experiments in terms of class distribution in this research. In these experiments, 96.2% accuracy for normal and pneumonia classes and 93.6% accuracy for viral and bacterial classes were the best performances. Finally, they utilized Grad-CAM to visualize the regions of the images that contributed most to the model's predictions.

Rahman et al. (2020) performed a transfer learning technique using four different pretrained convolutional neural network (CNN) models, such as AlexNet, ResNet-18, DenseNet-201, and SqueezeNet. All these trained models used an OCT-CXR dataset that included 5,237 chest X-ray images. In this research, the authors reported three different experiments in terms of dataset class combinations: case-1: normal and pneumonia; case-2: normal, bacterial pneumonia and viral pneumonia; and case-3: bacterial and viral pneumonia. The accuracies of these experiments were 98% (case-1: DenseNet), 95% (case-2: DenseNet), and 93.3% (case-3: DenseNet). Moreover, they also incorporated LIME to better explain the predictions of the model.

Hashmi et al. (2020) applied a transfer learning approach to classify binary classes as normal or pneumonia. Several data augmentation techniques simulate the regeneration of data images. The OCT-CXR dataset was utilized to retrain several experimental deep models (basic ResNet-18, Xception, Inception-V3, DenseNet-121, and MobileNet-V3 models and weighted mentioned renown models). This research concludes that renowned basic deep models with weightage perform better (accuracy: 98%) than other normal deep learning models (accuracy: 96–97%) by employing a data augmentation technique that reduces data overfitting. Additionally, they unveiled the model's reasoning by incorporating a technique called SHAP to explain the predictions of the model.

Ayan and Ünver (2019) performed an addition to this research to diagnose pneumonia by applying two CNN models, namely, VGG-16 and Xception, on the OCT-CXR dataset. The VGG-16 model achieved 87% accuracy, which was better than that of the Xception model (82%), and the confusion matrix showed that both models had their own significance against the data. The Xception network is more efficient at detecting pneumonia cases than is the normal network, while the Vgg-16 network focuses on normal patients. In addition, they elaborated upon the model and features by employing the saliency map technique to visualize the regions of the images that contributed most to the model's predictions.

Alshmrani et al. (2023) proposed a deep learning architecture for the identification and classification of multiple classes of lung diseases, such as pneumonia, lung cancer, and COVID-19. For multiclassification, they utilized large amounts of data for deep learning classification, including the OCT-CXR dataset. The proposed technique uses the ensembled methodology of the VGG-19 and custom-CNN models. This ensembled technique achieved the best performances−96.48, 97.56, and 93.75%—for accuracy, precision and sensitivity, respectively. They also provided explanations by utilizing a technique called attention maps to visualize the regions of the images that the model is paying attention to.

#### 5.2.3 Ensemble features/models

The ensemble approach is a technique used to ensemble multiple features from various trained models or deep models on the same dataset and provides better results than other techniques. In this domain, Chouhan et al. (2020) proposed a technique in which two or more deep learning algorithm models (AlexNet, ResNet-18, Inception-V3, DenseNet-121 and GoogleNet) were used for classification of images in the OCT-CXR dataset. After embedding, a voting classifier is employed to predict the best relevance class. The ensembled models outperform other state-of-the-art methods and achieve a 96% accuracy score. For explainability, they also employed a technique called integrated gradients to explain the predictions of the model.

Togaçar et al. (2020) applied three different pretrained deep models (AlexNet, VGG-16, and VGG-19). In the training process, every model obtained 1,000 features from every image in the dataset. Furthermore, the minimum redundancy maximum relevance algorithm was used to reduce the feature space to 100. On the basis of the combination of these features, a linear discriminant analysis classifier was trained and tested on a 99% binary classification accuracy on the OCT-CXR dataset. The authors also used a feature importance technique to explain the importance of different features in the model's predictions.

Ukwuoma et al. (2023) presented a hybrid workflow based on fused capabilities of convolutional networks and the transformer encoder (TE) mechanism. This ensembled learning technique employed to extract meaningful features from the dataset X-ray input images in two different ways: ensemble-A (i.e., GoogleNet, DenseNet201, and VGG16) and ensemble-B (i.e., Xception, DenseNet201, and InceptionResNetV2). Whereas, the TE is built following the scheme of self-attention structure considering multilayer perceptron (MLP) for accurate disease identification. The proposed whole designed pipeline underwent training in two fashions binary and multi-class classification. On test, end-to-end hybrid learning model resulted in 99.21% classification performance for both overall accuracy and F1-score in the binary classification task, while 98.19 and 97.29% scores for overall-accuracy and F1-score respectively have been expressed in the multi-classification task. In addition, they also showed the visual results by employing XAI, LIME and attention maps algorithms, to explain the predictions of the model.

## 6 Comparative analysis

In comparative analysis, we have evaluated the performance of various conventional machine learning and deep learning models, including customized CNN, transfer learning, and embedded models, utilizing different feature extraction techniques. For a more enhanced overview, different research works have been clustered into meaningful groups.

The performance metrics listed in this review are recommended methods for determining each model's performance. However, we have provided all the relevant measurements as shown in Table 3. To facilitate comparison, all the studies previously described were evaluated based on accuracy, with additional performance measures noted where available.

**Table 3:** Summarizing related studies that employ machine and deep learning methods where metrics are accuracy (Acc), specificity (Spe), precision (Pre), and recall (Rec), all presented as percentages; additionally, “N” denotes not present, respectively.

**References**	**Method**	**Features extractor**	**Classifier**	**Acc**	**Spe**	**Pre**	**Rec**	**XAI**
Oliveira et al. (2008)		Haar Wavelet Transforms	KNN	97	90	N	100	N
Sousa et al. (2013)	Machine Learning	Coefficient of Variation, Correlation, Entropy, Standard Deviation, etc., based on Haar wavelet	SVM, KNN, and Naïve Bayes	77, 70, and 68	N	N	N	N
Yao et al. (2011)		Techniques	Mean, variance, energy and correlation from correlation matrix, etc.	SVM	80	N	N	N	N
Stephen et al. (2019)			4-layers CNN		94	N	N	N	N
Jain et al. (2020)			CNN Model		92	N	N	98	N
Labhane et al. (2020)		Inception-V3, VGG-19, VGG-16, Customized-CNN		97	N	98	97	N
Siddiqi (2019)	Customized CNN	18-layers Convolutional	SoftMax Layer	94	86	92	99	N
Liang and Zheng (2020)				Custom 49- layers CNN, VGG-16, DenseNet-121, Inception-V3, and Xception		91, 74, 82, 85, and 88	N	89, 72, 79, 92, and 86	97, 95, 96, 84, and 97	N
Saraiva et al. (2019)				Custom CNN and MLP		92 and 94	92 and 94	92 and 94	92 and 94	N
Yi et al. (2023)				Custom CNN		96	99	N	94	N
Akbulut (2023)				LSTM + Attention-CNN in one model		96	98	94	94	N
Kiliçarslan et al. (2023)				Custom CNN		95	N	N	N	N
Rajaraman et al. (2019)		VGG-16		96	96	98	96	Grad-CAM
Rahman et al. (2020)		AlexNet, ResNet-18, DenseNet-201, and SqueezeNet		98, 95, and 93	97, 94, and 97	97, 95, and 93	99,96, and 93	LIME
Hashmi et al. (2020)	Transfer Learning	Original and Weighted (ResNet-18, Xception, Inception-V3, DenseNet-121, and MobileNet-V3)		96-97 and 98	N	98	99	SHAP
Ayan and Ünver (2019)			VGG-16 and Xception		87 and 82	91 and 76	87 and 84	82 and 85	Saliency maps
Alshmrani et al. (2023)		VGG-19 and Custom CNN		97	N	98	94	Attention maps
Chouhan et al. (2020)		AlexNet, ResNet-18, Inception-V3, DenseNet-121 and GoogleNet	Voting Classifier	96	N	93	100	Integrated gradients
Togaçar et al. (2020)	Ensemble Model	AlexNet, VGG-16 and VGG-19	Linear Discriminant Analysis Classification (Merged features)	99	99	99	100	Feature importance by model's predictions
Ukwuoma et al. (2023)			DenseNet201, VGG16, and GoogleNet	Multilayer Perceptron	97	97	97	97	LIME and attention maps

In the groups, ML group depicts that Oliveira et al. (2008) achieved the best 97% accuracy by employing the k-NN classifier on numerous extracted features. With respect to customized neural networks, Labhane et al. (2020) achieved ~97% accuracy, with the best results obtained with all the customized and pretrained models. Using the pretrained transfer learning technique, Rahman et al. (2020) and Hashmi et al. (2020) achieved 98% accuracy by employing numerous pretrained models by comparing among them. In the last group, Togaçar et al. (2020) achieved 99% accuracy by employing transfer learning and the ensemble feature technique. Overall, we found that Togaçar et al. (2020) performed very well, with 99% accuracy, by using transfer learning and the ensemble feature technique. This shows that transfer learning with an ensemble method is an efficient and effective technique for learning and representing better visual features of images.

## 7 Progress and challenges in chest research applications

As AI continues to advance over time, it has become increasingly convenient to develop improved algorithms, reducing the reliance on manually crafted features for radiographic images. Researchers have made significant strides in creating numerous algorithms designed to automatically extract features and employ machine/deep learning techniques. The primary benefit of these methods lies in their enhanced performance, particularly in the detection and classification of various chest diseases using deep learning algorithms. Many recent studies have harnessed convolutional neural networks (CNNs) to augment early-stage detection by learning intricate patterns, thereby helping doctors comprehend these complex situations.

However, a notable challenge is the lack of practical applicability, which hinders the widespread use of these highly efficient algorithms. There is a pressing need to bridge the gap between related research and wider public and medical communities by creating user-friendly interfaces on well-performing models. Moreover, there is a deficiency in training resources because of the limited tools available, leaving many users uncertain about how to effectively utilize these tools in middle- and low-income regions for early diagnosis and mitigation of chest and related diseases.

In addition, while the number of studies on the detection of pneumonia using machine learning/deep learning is growing, the number of works that focus on explainability is still relatively limited. This is a significant gap in the literature, as explainability is crucial for understanding how models make decisions and for building trust in their results.

## 8 Conclusion

The detection of lung diseases from medical images poses significant challenges for radiologists, even for experienced professionals, due to the intricacies of these diseases and the time-intensive nature of the diagnostic process. However, artificial intelligence (AI) has emerged as a promising solution for addressing these challenges. Artificial intelligence (AI), particularly through machine learning and deep learning techniques, has revolutionized the field of medical research, replaced traditional handcrafted methods and significantly improved diagnostic accuracy. In this review, we have presented a vast amount of research on chest disease classification using four different standard radio imaging datasets of lungs. Among the datasets considered, the CXR dataset is the most commonly used dataset among the reviewed works compared to the ChestX-ray14, JSRT, and MC datasets. For classification, different image preprocessing and feature extraction techniques have been employed to train different machines and deep learning algorithms. Overall, it has been found that transfer learning techniques with ensemble models/features result very well-compared to machine learning, custom-designed deep models, and transfer learning models. Additionally, our work highlights the importance of XAI, and its usage in limited works can unveil hidden reasoning. Ultimately, this comprehensive review will not only benefit researchers related to lung diseases but also increase the interest of researchers working on images, machine learning, machine vision, deep learning, and other related areas.
